# Insidious Destroyers

**Published:** 1883-10

**Authors:** 


### INSIDIOUS DESTROYERS.

THERE are dangers that beset us on every side ; some of these we avoid instinctively. Thus we step aside from a passing vehicle or avoid going too near a precipice. But there are unseen dangers, concealed pitfalls, to which we are constantly exposed and that count thousands of unsuspecting victims. We put aside, for the present, the consideration of the vices and crimes of mankind, those destroyers that draw the helpless and the innocent along with the guilty into the maelstrom of death.

Ignorance and indifference in the selection of foods and other articles, errors in diet, in dress and in the general care of the health, are evils that pervade every class of society.

Our bodies are built up, and the machinery of life is kept in motion by what we eat and drink. If our food is appropriate and wholesome, and taken in proper quantities, health of body and vigor of mind result ; if, on the contrary, the food is bad or insufficient, the body suffers in consequence.

Greed of gain often destroys the consciences of men. Adulterations of the necessaries of life as well as its luxuries are common. That sort of thing the perpetrators would like to have regarded as evidence of business enterprise and shrewdness rather than in its true light of heartless rascality that merits severer punishment than more easily detected crimes. The lapse of four thousand years has not effaced the crime of Pharaoh for the slaughter of the "Innocents." Fanaticism, not *avarice*, was the motive for this cruelty of the

Egyptian king, and it was confined to the male children of the Hebrew race. But the manufacturers of adulterated articles seem to have no regard for sex, age, race or country. The natural craving of children for sweets is taken advantage of, and substances permanently detrimental to the health of any one, and particularly of children, are manufactured and sold in vast quantities as candies. Hundreds of tons of white earth or kaoline, are used every year in the adulteration of candies and various foods, to say nothing of other hundreds of tons of glucose that are supplied for the same purpose, besides quantities of poisonous pigments and essential oils. There are candies that are pure; some such are usually found in candy shops.

Since children require sweets, a good plan is to give them granulated sugar. If you wish to see a picture of perfect enjoyment, give a child half a teacupful of granulated sugar and a teaspoon.

There can be no controversy as to the fact that there is some secret agent connected with modern methods of living that is responsible for the increasing prevalence of diseases of the kidneys among adults ; and so long as the actual cause is involved in doubt, it is prudent to discard from our homes all suspected preparations, intended for food, or that enter into foods.

It is but a few years since the whole country was startled by the revelations of chemists and physicians in regard to the deleterious effects of alum when taken into the stomach ; and to "baking powders" containing *alum* were attributed certain affections of the stomach, liver and kidneys that, it must be admitted, have increased in a ratio commensurate with the increased consumption of adulterated "baking powders." But are there not dangers lurking in baking powders that are *claimed* to be pure ? It is a fact well known to chemists and physicians that deadly poisons may be compounded from substances that in themselves are inert and harmless. It would seem the part of simple prudence to avoid "baking powders" that contain several chemicals in combination. For example, a baking powder containing four or five chemicals mingled together, may be as harmless as any other, but still we should not care to risk the ultimate results of such a mixture on the sensitive organs of the body with which it would come in contact, as there is no need of such risk.

A "baking powder" made from *pure* CREAM OF TARTAR, *pure* BICARBONATE OF SODA and the requisite quantity of flour or starch, as a preservative, without suspicion of any other ingredient, is, in our opinion, as wholesome as yeast for all uses to which it is adapted. We know that Cleveland's Superior Baking Powder is so made. We purchase the same for use in our own family of the manufacturers, Cleveland Brothers, Albany, N. Y.

